# Quercetin as a Novel Therapeutic Approach for Lymphoma

**DOI:** 10.1155/2021/3157867

**Published:** 2021-08-02

**Authors:** Saiedeh Razi Soofiyani, Kamran Hosseini, Haleh Forouhandeh, Tohid Ghasemnejad, Vahideh Tarhriz, Parina Asgharian, Željko Reiner, Javad Sharifi-Rad, William C. Cho

**Affiliations:** ^1^Clinical Research Development Unit of Sina Educational, Research, and Treatment Center, Tabriz University of Medical Sciences, Tabriz, Iran; ^2^Student Research Committee, Shiraz University of Medical Sciences, Shiraz, Iran; ^3^Department of Molecular Medicine, Faculty of Advanced Medical Sciences and Technologies, Shiraz University of Medical Sciences, Shiraz, Iran; ^4^Molecular Medicine Research Center, Biomedicine Institute, Tabriz University of Medical Sciences, Tabriz, Iran; ^5^Department of Pharmacognosy, Faculty of Pharmacy, Tabriz University of Medical Sciences, Tabriz, Iran; ^6^Drug Applied Research Center, Tabriz University of Medical Sciences, Tabriz, Iran; ^7^Department of Internal Medicine, University Hospital Centre Zagreb, School of Medicine, University of Zagreb, Zagreb, Croatia; ^8^Phytochemistry Research Center, Shahid Beheshti University of Medical Sciences, Tehran, Iran; ^9^Department of Clinical Oncology, Queen Elizabeth Hospital, Kowloon, Hong Kong

## Abstract

Lymphoma is a name for malignant diseases of the lymphatic system including Hodgkin's lymphoma and non-Hodgkin's lymphoma. Although several approaches are used for the treatment of these diseases, some of them are not successful and have serious adverse effects. Therefore, other effective treatment methods might be interesting. Studies have indicated that plant ingredients play a key role in treating several diseases. Some plants have already shown a potential therapeutic effect on many malignant diseases. Quercetin is a flavonoid found in different plants and could be useful in the treatment of different malignant diseases. Quercetin has its antimalignant effects through targeting main survival pathways activated in tumor cells. *In vitro*/*in vivo* experimental studies have demonstrated that quercetin possesses a cytotoxic effect on lymphoid cancer cells. Regardless of the optimum results that have been obtained from both *in vitro*/*in vivo* studies, few clinical studies have analyzed the antitumor effects of quercetin in lymphoid cancers. Thus, it seems that more clinical studies should introduce quercetin as a therapeutic, alone or in combination with other chemotherapy agents. Here, in this study, we reviewed the anticancer effects of quercetin and highlighted the potential therapeutic effects of quercetin in various types of lymphoma.

## 1. Introduction

The lymphatic system has important functions such as maintaining tissue fluid homeostasis, initiating humoral and cellular immune responses, and transporting intestinal lipids to the blood [1]. According to the classical theory of metastasis, malignant cells that reach the sentinel lymph nodes may spread further into the distal lymph nodes, and subsequently form organ metastases [2]. Traditionally, four treatment methods including surgery, radiotherapy, chemotherapy, and immunotherapy are used alone or in combination to treat patients' malignant diseases [3, 4]. Chemotherapy is used as a common approach for the treatment of these patients; however, drug resistance is the key reason for decreased effectiveness of chemotherapy and occurs due to adaptation of cancer cells to chemotherapy agents [5]. The development of analytical tools, genome extraction, engineering strategies, and microbial science have opened a new window on the sciences of pharmacotherapy and cancer therapy. Given this development, in relation to cancer and some infectious diseases, their natural products and structural analogues are of great help to researchers in the field of pharmacology [6]. Producing substances which are capable of killing malignant cells without being toxic is important and has attracted increasing interest. In recent years, it has been shown that some substances derived from plants can have beneficial effects on malignant cells [7–10]. Flavonoids have been the most studied. They interact in specific stages of the carcinogenesis process to prevent cell proliferation and consequently cause apoptosis [11–15]. Quercetin (3,3′,4′,5,7^″^ penta-hydroxyl flavones) is a polyphenolic flavonoid found in apples, red grapes, onions, raspberries, honey, cherries, citrus fruits, and green leafy vegetables [16].

In recent years, several studies have demonstrated that quercetin has different biological effects [17], including antiviral, cell-cycle modulation, inhibitory effects on antioxidant angiogenesis, anticancer, apoptosis-inducing induction, and C-blocking protein kinase [18–20]. It was suggested that chronic daily intake of quercetin might prevent some types of malignant diseases as well as lymphoid cancers. Several pathways have been identified that are affected by quercetin in various malignancies [21, 22]. Here, for the first time, we reviewed the anticancer and therapeutic effects of quercetin in different types of lymphoid cancers.

## 2. Lymphomas and Possible Causes of Increased Lymphoma

The lymphatic system is essential for immune response, maintaining tissue fluid balance, transporting antigen and antigen-presenting cells (APCs) to the lymph nodes, and transporting lipids absorbed from the intestine into the blood. Therefore, disorders of the lymphatic system can cause lymphedema, compromise local immune response, and cause intestinal malabsorption [23]. The lymphatic system includes the lymph nodes, spleen, thymus, and bone marrow. Lymphomas can affect all of them as well as other organs. Lymphoma is a malignant disease of the lymphatic system. There are several types of lymphoma: Hodgkin's lymphoma (formerly called Hodgkin's disease) and non-Hodgkin's lymphoma [24]. Causes and risk factors that increase the susceptibility to lymphoma include age (people over 60), gender (more prevalent in women than men), obesity, ethnicity (e.g., high percentage within white Americans), exposure to chemicals or radiation (such as pesticides and nuclear radiation), infections (such as HTLV-1 virus, *H. pylori*, hepatitis C, and EBV), autoimmune diseases (such as RA and celiac disease), immune deficiencies (such as AIDS), or receiving immunosuppressive drugs [25–27]. The role of the lymphatic system in the progression of metastasis of malignancies has attracted an increased interest in the last 15 years [28]. The lymphatic system also plays a critical role in cancer progression, because the metastatic cancer cells can spread through the lymph vessels to the lymph nodes [23]. The most common sites of solid tumor metastases are lymph nodes. Their presence indicates a poor prognosis and usually suggests the need for systemic treatment in patients with malignant disease. On the other hand, the presence of cancer cells in the lymph nodes may reflect the ability of the primary tumor to metastasize; however, their actual presence in the lymph nodes may be inconclusive [29, 30]. This reasoning indicates that lymph node metastases need to be treated for prevention of distant metastasis and ultimately eradication of the disease [31–33]. The metastases in lymph nodes might indicate whether there is a possibility of further expansion of the malignancy in lymph nodes or whether distal metastases have already occurred [34]. Lymph node metastasis is thought to be regulated in two stages; the first one being the uptake of malignant cells into the lymph vessels and the second one a successful penetration of these cells into lymph nodes [35]. Because of the lack of functional lymph vessels in tumor cells, interstitial fluid pressure increases and the lymph flow to the lymph nodes changes, which causes the tumors to drain [36]. Tumor-induced VEGF-C and VEGF-D increase the proximal contraction of lymphatic vessels [37]. The expansion of malignant cells to the lymph nodes is decreased when tumor-induced lymph vessel regeneration is prevented [36, 38–40].

## 3. Quercetin and Its Antitumor Effects

**Figure 1:**
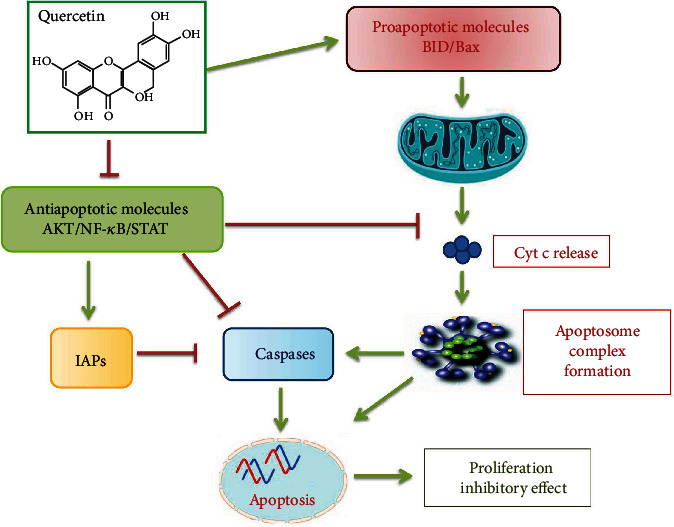
Suggested model for quercetin effects on lymphoma cells. Quercetin inhibits cell growth by apoptosis induction via inhibitory effects on antiapoptotic signaling molecules and proapoptotic protein induction which cause activation of mitochondrial-mediated caspase activation and apoptosis [43, 51, 59, 60].

Despite great advances in the treatment of malignant diseases, they are still recognized as life-threatening diseases. However, the effects of chemotherapy which is used as a common treatment for many malignant diseases are limited because of resistance to this type of therapy and serious adverse effects. Today, natural compounds such as quercetin are recognized as an important factor in malignant disease prevention and treatment because of their effects, high therapeutic potential, and low toxicity [41]. Quercetin, as a lipophilic compound, can cross cell membranes and initiate several intracellular signaling pathways. Quercetin's unique characteristic includes its dual function as a peroxidant or antioxidant [42]. It seems that quercetin can act as a potential antiproliferation and antimalignant as well as apoptosis inducer (Figure 1) [41, 43]. The mitochondrial-mediated pathway has been suggested as the main mechanism by quercetin to induce apoptosis [44–46]. It also induces apoptosis by increasing proapoptotic molecules such as Bax, caspase-3, caspase-9, p53, or decreasing of antiapoptotic substances [47]. Quercetin prevents the cell cycle by reducing D1/Cdk4 and E/Cdk2 and regulating p21, which ultimately stops the G1 cell cycle [48]. Quercetin binds directly to tubulin and eliminate the polymerization of cell microtubules, which causes stopping of the cell cycle [49]. It seems that quercetin interferes with a large variety of molecules involved in the cell cycle. The complex of PI3K/AKT/PKB is a pathway involved in different processes such as regulation of cell survival, cell cycle, growth progression, and carcinogenesis [50]. This pathway can be targeted with quercetin in different types of malignancies and cause induction of apoptosis, inhibition of cancerogenesis, and development of malignancy [51]. Numerous studies have reported that quercetin causes phosphorylation and stabilization of p53 levels. p53 is considered to be a key molecule in the proapoptotic and antimalignant effects of quercetin [52]. p53 has an antioxidant effect through regulating several genes, including aldehyde dehydrogenase ALDH4A1, Mn-SOD2, microsomal GSH homologous transferase PIG12, Gpx1, and catalase [53–55]. A decrease in p53 sensitizes cells to H_2_O_2_ damage, which causes decreased viability and increased apoptosis and DNA oxidation. The function of P53 in malignant cells is significantly blocked by poor regulation of its genes [56]. Increased ROS as oxidative stress causes DNA damage and mutations. Quercetin can reduce ROS by electron donation that leads to reduce ROS-mediated DNA damage [57, 58]. According to the results of related studies, it could be concluded that quercetin has an important role to treat and control malignancies. Regulation of cell-cycle-related proteins, antioxidant effects, induction of apoptosis, and induction of cell-cycle arrest are some of the mechanisms by which quercetin has its effects in the treatment and inhibition of malignancies.

## 4. Molecular Targets of Quercetin in Malignant Cells

Malignant diseases have an abnormal activation of various survival signaling pathways, e.g., a different antiapoptotic protein expression [61]. Aberrant activation of signaling pathways such as serine-threonine kinase, activator protein 1 (AP-1), AKT kinase/protein kinase B (AKT/PKB), nuclear factor kappa B (NF-*κ*B), mitogen-activated protein kinase (MAPK), androgen receptor, Raf/Ras pathways, and estrogen receptors are the key mechanisms that can cause tumorigenesis [62, 63].

**Figure 2:**
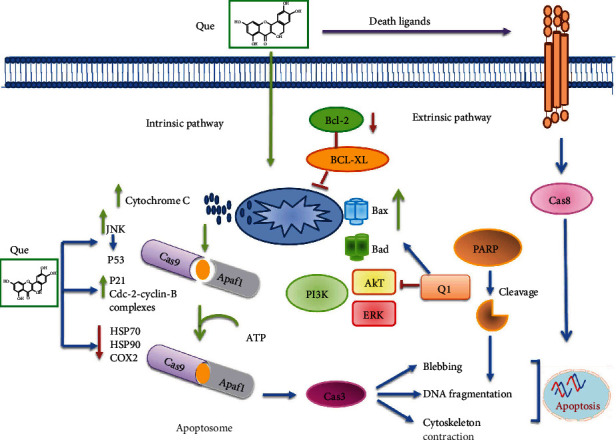
A view of the effect of quercetin on the expression level of genes involved in apoptosis pathway [65–68].

Tyrosine kinases like Jak and Src kinases are activated in malignant cells [64]. These signaling transductions are essential for normal cell proliferation and physiological function. In malignant cells, deregulated expression of these pathways results in abnormal cell growth and apoptosis resistance. Several studies have been conducted to explore the strategies of targeting the overactivated molecules involved in survival pathways to induce apoptosis in malignant cells [16]. It has been shown that quercetin inhibits cell growth by cell-cycle arrest and induces apoptosis in different malignant cells by targeting different molecular pathways such as activation of NF-*κ*B and signal transducers/activators of transcription STAT3 (Figure 2) [65–68]. It can also downregulate the expression NF-*κ*B, Bcl-2, cyclooxygenase 2 (COX-2), matrix metalloprotease 9 (MMP-9), tumor necrosis factor (TNF), cyclin D1, and adhesion molecules [69–73]. NF-*κ*B is one of the important transcription factors identified in the nucleus of B lymphocytes and plays a critical role in regulating the function of the human immune system. This transcription factor controls the expression of many genes and has been linked to inflammatory, neurological, and cancer diseases [74]. Numerous studies have shown that NF-*κ*B activation is associated with some lymph node diseases, including large cell lymphoma, Burkitt's lymphoma, diffuse large B-cell lymphoma, fibrosarcoma, Hodgkin's lymphoma, mammary carcinoma, and mantle cell lymphoma melanoma [75].

Several research indicated that the chemopreventive/chemotherapeutic effects of quercetin are based on its concentration. At low doses, quercetin acts as an antioxidant. However, at high doses, quercetin has effects of a prooxidant and prompts chemotherapeutic effects. The antitumor effects of quercetin are related to its potential in reducing tumor growth, inducing apoptosis, promoting cell-cycle arrest, and suppressing mitotic processes, which are performed by modulating cyclin, proapoptotic, PI3K/AKT, and mitogen-activated protein kinase (MAPK) molecular pathways (Table 1) [76]. Quercetin also downregulates p-ERK1/p-ERK 2 in the multiple myeloma (MM) cell lines which results in inhibition of MAPK pathway activation and suppression MM cell proliferation.

## 5. Quercetin and Lymphomas

### 5.1. Multiple Myeloma and Quercetin

Multiple myeloma is characterized by accumulation of plasma cells in the bone marrow and an increased monoclonal protein concentration in blood and/or urine [89]. The effects of quercetin on multiple myeloma cells have been reported in several studies [85, 86, 90]. The deregulation of apoptosis in plasma cells plays a key role in the pathogenesis and chemoresistance of multiple myeloma. Quercetin inhibits IQ motif-containing GTPase activating protein 1 (IQGAP1) expression in multiple myeloma cells. IQGAP1 activates growth factor-mediated activation of extracellular signal-regulated kinase (ERK) and mitogen-activated protein kinase (MAPK)/ERK kinase (MEK). The RAS/MEK/ERK signaling pathway plays a key role in MM pathogenesis [86]. Xu et al. demonstrated that quercetin activates the apoptosis-related proteins and induces cell-cycle arrest in MM cell lines. It also upregulates the apoptosis-related proteins including caspase-3, 8, and 9; upregulates PARP; and inhibits Bcl-2 expression. Quercetin induces the expression of P53, P21, and P27 and reduces the phosphorylation levels of p-ERK and p-A KT in MM cell lines [85].

### 5.2. Quercetin and T-Cell Lymphoma

T-cell lymphomas are a group of diseases characterized by clonal expansion and dysfunction of T-cells. T-cell lymphomas have been classified into two groups: cutaneous TCL (CTCL) and peripheral TCL (PTCL) [91, 92]. The PI3K/AKT pathway is the main target in antimalignant therapy and PI3K as a lipid kinase plays a key role in malignant cell transformation. PI3K is involved in regulating cell growth, survival, apoptosis, and angiogenesis. It also activates AKT which has a key function in both physiological and pathological signaling pathways. The antimalignant effect of PI3K is based on inhibition in hematological malignancies via the blocking of survival signaling pathways within malignant and nonmalignant cells along with controlling cytokine secretion and activating the immune response. Maurya et al. indicated that in human leukemic cell lines, quercetin can inhibit upregulation of AKT1/2 and tensin homolog (PTEN) pathways by reducing PI3K/AKT activity and suppressing the proliferation in human leukemic cell lines. AKT induces cell survival by phosphorylating/inhibiting Bcl-2-associated agonist of cell death (BAD) which promotes cell death and apoptosis by its dephosphorylation. Therefore, BAD phosphorylation stimulates cell survival. Quercetin attenuates the PI3K/AKT pathway and prevents survival signals in lymphoma growth [88].

### 5.3. Quercetin and Large B Lymphomas (Hodgkin and Non-Hodgkin)

Another type of lymphoma is the B-cell lymphoma, which is classified into Hodgkin's and non-Hodgkin's lymphomas. According to the World Health Organization (WHO), B-cell lymphomas are classified into five major categories: diffuse large B-cell lymphoma (DLBCL), follicular lymphoma, marginal zone B-cell lymphoma (MZL) or mucosa-associated lymphatic tissue lymphoma (MALT), small lymphocytic lymphoma (SLL), and mantle cell lymphoma (MCL) [93]. Aggressive lymphomas require serious treatment. On the other hand, this type of malignancy therapy depends on the type of lymphoma and the stage and grade of the disease. For example, slow-growing lymphomas can be treated with radiation therapy, but the aggressive type has to be treated with chemotherapy in addition to radiation therapy [94, 95]. Chemotherapy and radiation therapy have many adverse effects. Therefore, some researchers have tried to use therapies with fewer side effects, such as herbal compounds. One of these plant substances is quercetin, which is rich in polyphenols and flavonoids and has been shown to have anti-inflammatory, antimalignant, and antioxidant (free radical scavenger) effects [96–98]. Fil'chenkov et al. investigated the antiapoptotic effects of quercetin and resveratrol in vitro on B-cell lymphoma cell lines (Namalawa). Using flow cytometry and 1H NMR spectroscopy, they found that these two substances activated caspase-3 apoptotic cycles and increased hypodiploid cells as well as the number of motile lipid domains. Thus, the combination with quercetin causes time-dependent arrest of cells in the G2/M phase [79]. Lee et al. studied the U937 lymphoma cell line and found that the main mechanism of inhibition of lymphoma cell growth is related to cell-cycle arrest in the G2/M phase and induction of the caspase-mediated apoptotic process. They found that quercetin stabilized U937 cells in the G2/M phase, which in turn increased cyclin B protein levels and decreased cyclin D, E, E2F1, and E2F2 levels, and vice versa. Quercetin stimulates cell death by modulating Bcl-2 and Bcl-xl without altering the expression of Bcl-2 and Bcl-xl proteins, and this action is mediated by caspase-3. Finally, quercetin makes time-dependent accumulation of U937 cells in the G2/M phase in which arrest occurs and increases the time-dependent cell population in the sub-G1 phase [77]. Frankenfeld et al. studied the association of dietary flavonoid intake with a reduced risk of developing non-Hodgkin's lymphoma (NHL). They found that consuming more flavonols such as quercetin, epicatechins, anthocyanidins, and proanthocyanidins reduces the risk by 47% in the highest quarter compared to the lowest (95% CI: 31%, 73%), and that the more flavonols were consumed, the lower was the risk of non-Hodgkin lymphoma [99]. Kawahara et al. studied lymphoma and leukemia cell lines, especially Daudi (Burkitt's lymphoma) and TMD-8 (diffuse large B-cell lymphoma), and found that cyclopamine and quercetin inhibited cell line growth and stimulated apoptosis. Cyclopamine targets the Hh signaling protein called Gli1 and reduces its expression. Quercetin reduces the expression of Notch1 protein and its active component in the DND-41 lymphocytic T leukemia cell line. They concluded that these natural substances could be used as targeted treatment for chemotherapy-resistant lymphoma and leukemia [78].

Jacquemin et al. found that quercetin combined with TRAIL therapy could be beneficial for malignant diseases such as non-Hodgkin's lymphoma (NHL). They found that pretreatment of malignant cells with 20 *μ*M quercetin for 24 hours overcomes the resistance of lymphoma cell lines to tumor necrosis factor-related apoptosis-inducing ligand- (TRAIL-) stimulated cell death. This compound completely activates caspase-3, and then it activates caspase-8 and caspase-9. Using flow cytometric analysis, they have shown that quercetin pretreatment prevents the expression of TRAIL receptors in lymphoma cell lines such as RL and VAL. In these cells, quercetin increases the expression of caspase-10 following TRAIL stimulation, but it is not needed for the reversal of apoptosis. Quercetin sensitization is independent of caspase-10 and the regulation of TRAIL-RISC complex formation. Quercetin reactivates the caspase-independent mitochondrial pathway by reducing the expression of survivin and Mcl-1, except p53 [80]. Rituximab is a chimeric monoclonal antibody against the CD20 protein on the surface of B cells and causes cell death after binding of the ligand to the receptor. This drug is used in the treatment of non-Hodgkin's lymphoma (NHL), chronic myelocytic leukemia (CLL), autoimmune diseases such as rheumatoid arthritis (RA), and skin lesions caused by the EBV virus. Li et al. studied the effects of monoclonal antibody in combination with quercetin on DLBCL lymphoma cell lines (WUS-DLCL-2 and SUDHL-4). They found that by using the cell viability method, these cell lines are sensitive to 20 *μ*M quercetin; furthermore, by using flow cytometry, quercetin induces apoptosis in these cell lines. The amount of 20 *μ*M quercetin together with 5 *μ*g/ml of antibody increased the sensitivity of the cell lines to the rituximab antibody. They also found that antibody-induced apoptosis was amplified by quercetin on the cell lines, and the quercetin-antibody complex inhibited the p-STAT3 pathway (involved in the regulation of Mcl-1, survivin and Bcl-x1 genes) because the expression of these genes was significantly reduced [81].

In addition to the STAT3 signaling pathway, the PI3K/AKT/mTOR and Wnt/*β*-catenin pathways can be inhibited by natural products such as quercetin. These pathways cause highly aggressive B-cell lymphomas, such as primary effusion lymphoma (PEL). The Granato research team found that quercetin induces the death of the BC1, BC3, and BCBL1 cell lines in a dose-dependent manner by increasing the number of G1 events and causing cell death by PARP cleavage. They also found that quercetin inhibited PI3K and mTOR kinase and reduced the Wnt activation pathway, which eventually caused cell death to occur by inhibiting PI3K/AKT/mTOR signaling. mTOR was less phosphorylated, and quercetin reduced AKT phosphorylation at the Ser473 position. Quercetin also decreased prosurvival molecules downstream of these pathways, such as c-FLIP, cyclin D1, and c-Myc, and it reduced nuclear localization of p65 NF-*κ*B. They showed that protein phosphatase inhibitors neutralized dephosphorylation of tyrosine STAT3 by quercetin in the BC3 cell line, and since it has two-way communication between these pathways, it seems that quercetin reduces the release of the IL-6 and IL-10 cytokines. According to them, quercetin regulates the autophagy process by inhibiting the mTOR pathway and the STAT3 pathway and reducing the expression of FADD-like IL-1*β*-converting enzyme- (FLICE -) inhibitory protein (c-FLIP) and viral FLICE-Inhibitory Proteins (v-FLIPs) in the cell lines. In addition, the survival of BC3 and body-cavity-based lymphoma (BCBL1) cells is reduced by the quercetin-bortezomib complex and quercetin increases the activity of this proteasome inhibitor. Following the activation of T_h_ CD4^+^ cells, the expression of HLA II molecules (HLA-DR) in cells increases and the expression of HLA I decreases, and quercetin induces exposure to calreticulin on the surface of malignant cells [59].

Salazar et al. studied the *HIGD2A* gene (involved in cell survival under hypoxia) and its role in diffuse large B-cell lymphoma (DLBCL) by modulating the expression of this gene in mice. Using RT-qPCR analysis, they found that exposure to 50 mg/ml quercetin caused the expression of this gene in the bone marrow tissue (reduced hypoxia) of lymphoma mice. In addition, it increased in the liver and spleen tissue (increased hypoxia) due to the rapid proliferation of tissue, while in healthy mice, the expression of this gene in the bone marrow and spleen increased but it decreased in the liver. Finally, they found that *HIGD2A* gene expression increased in DLBCL cells and decreased in nodal marginal lymphoma (NMZL) [82].

### 5.4. Quercetin and Burkitt's Lymphoma

Another malignant disease of the lymphatic system is Burkitt's lymphoma. This type of malignancy is most often associated with B lymphocytes in the germinal regions [100]. Clinically, this malignancy is classified into three categories including the endemic or African variant in which the EBV virus infects children, the sporadic or non-African variant hosted by children, and the immune system defect-related variants that are often associated with HIV infection or in people who are immunocompromised [101–103]. These variants are often associated with mutations in c-Myc gene which is a regulator and protooncogene [104]. Risk factors for the disease include HIV/AIDS infection, posttransplantation immunosuppression, and malaria infection [105]. Treatment of this type of malignancy depends on the type of the disease. Nevertheless, the first line of treatment, depending on when the disease is diagnosed and whether the tumor is slow or fast growing, is the use of chemotherapy. Drugs which are used to treat Burkitt's lymphoma are cyclophosphamide, doxorubicin, methotrexate, rituximab, etc. In addition to chemotherapy, immunotherapy, bone marrow transplantation, stem cell transplantation, radiotherapy, etc., are also used for treatment [100]. Recently, herbal products with active ingredients have been used to treat this type of malignancy because they have less adverse effects. Based on their research on Raji cells (Burkitt's lymphoma cells), Okamoto et al. found that quercetin inhibited the stimulation of primary antigens (EA) by the EBV virus which was induced by 12-O-tetradcanoyl phorbol-13-acetate (TPA). They also found that doses of 25, 10, and 2.5 *μ*g/ml of quercetin inhibited 82, 74, and 32% of primary viral antigens, respectively. Finally, they concluded that natural inhibitory substances such as quercetin may also be beneficial in increasing lymphoma inhibition by TPA [106].

Inadequate functioning of the PI3K pathway is involved in the growth and development of a variety of malignancies. As mentioned earlier, quercetin inhibits the PI3K pathway. Lim et al. studied a subset of a quercetin called fisetin. They found that because PI3K has several isoforms and is expressed in the Raji cell line (gamma and delta isoforms are expressed more on these cells than alpha and beta), tPI3K*δ* can be targeted by fisetin followed by induction of apoptosis. They also showed that fisetin reduced the expression of anti-/proapoptotic molecules. Therefore, using the immune-blotting technique, 30 *μ*M concentration of fisetin reduced cIAP-1 and cIAP-2 protein expression. It also inhibited the mTOR pathway by inhibiting phosphorylation of the mTOR pathway (S2448) and its downstream targets, p70S6K and 4E-BP1. Following this process, apoptosis increased in Raji cells. 30 *μ*M of fisetin also increased the expression of *γ*H2A.X protein (a marker of cell DNA damage), which in turn indicated the proapoptotic activity of this substance on Raji cells [84].

As mentioned earlier, one of the causes of Burkitt's lymphoma is overexpression of the c-Myc gene and its translocation. Granato et al. demonstrated that quercetin generally reduced and inhibited the c-Myc expression and the PI3K/AKT/mTOR pathway and induced cytotoxicity against Raji, Akata, 2A8, Ramos, and BL-41 as Burkitt's lymphoma cells. The found that quercetin reduced tumorigenesis in BL-41 cells because 50 and 100 *μ*M concentrations of this substance decreased c-Myc expression in BL-41, Raji, Akata, and Ramos cells. By phosphorylating 70S6K and 4E-BP1, the mTOR pathway was inhibited. The effect of quercetin-mediated toxicity on BL-41 and Ramos cells was greater than on Raji and Akata cells. Western blot analysis showed that quercetin was involved in PARP fragment cleavage in apoptotic cell death. The decreased expression of the c-Myc protooncogene and inhibition of the PI3K/AKT/mTOR pathway leads to BL-41 cell death. It seems that quercetin reduces the expression of heat shock protein (HSP-70) followed by a decreased expression of c-Myc protein in BL-41 cells. Quercetin also affects HSP-90 expression and decreases HSP-70 expression in Raji and Akata cell lines. Quercetin stimulates the autophagy process in BL-41 cells because the lipidation of cells (autophagy markers) treated with this substance is increased and the induction of the autophagy process helps to reduce c-Myc expression in some cell lines [60].

### 5.5. Antimalignant Effect of Quercetin (Molecular Mechanisms)

Since it seems that quercetin has chemopreventive and chemotherapeutic effects in vivo and in vitro, it has been used as an antitumor drug. This natural product has antioxidant effects in small amounts and prooxidant effects in large amounts; therefore, it can be used as a chemotherapy drug by reducing malignant cell proliferation, apoptosis, cell-cycle arrest, etc., through various molecular pathways that have become more and more interesting [107–109].

### 5.6. Role in Cell Proliferation

One of the main roles of quercetin is to inhibit the growth and proliferation of many types of malignant cells, including lymphoma, prostate, cervix, lung, breast, and colon. Quercetin can cause cell-cycle arrest in the G2/M phase or G1 phase, depending on the type of cancer cell [77, 110, 111]. Cyclins as well as kinase-dependent cyclins (cdks) play a major regulatory role in the cell cycle [112, 113]. The degree of cell inhibition depends on the concentration of quercetin and has often been studied in vitro [114]. Quercetin-mediated cell-cycle regulation can be interceded by p21, p27, p53, and Chk2 proteins. Regulation of reduced expression of Cdk1 and cyclin B1 as well as pRb phosphorylation can be shown on many types of malignant cell lines [115, 116]. As mentioned before, Granato et al. have investigated cell biology, growth, and proliferation of B lymphoma cells (including BC1, BC3, and BCBL1 cell lines) at different concentrations of quercetin [59].

### 5.7. Role in Oxidative Stress

Oxidative stress is caused by an imbalance between oxidants (such as ROS) and antioxidants (such as detoxification systems) [117]. Quercetin increases the activity of antioxidants by increasing the glutathione (GSH) level. GSH as a donor is involved in the conversion of H_2_O_2_ to H_2_O by the enzyme superoxide dismutase (SOD). Quercetin stimulates GSH production [118]. The high activity of signaling pathways involved in cell survival, proliferation, and migration is directly related to oxidative stress. For example, ROS can activate the PI3K/AKT pathway, which in turn plays a crucial role in the growth and development of various malignancies [119]. According to Maurya et al., 300 *μ*M of quercetin inhibited the increase of ROS production, which in turn phosphorylated the AKT, PDK1, and Bcl-2 promoters, followed by downregulation. In Dalton lymphoma ascite (DLA) cells treated with H_2_O_2_, quercetin increased the level of PTEN protein [88].

### 5.8. Role in Autophagy

One of the natural cell mechanisms to remove unnecessary cell components is the process of autophagy, and due to this, the cell components are decomposed and recycled. This process helps to stimulate malignant growth by increasing the survival of malignant cells [120–122]. Several studies have shown that quercetin causes instability and does not induce autophagy in malignant cells. It also diminishes autophagy loss by reducing the stabilization of *β*-catenin and HIF-1*α* and inhibiting the phosphorylation of AKT, mTOR, and ERK [76]. The STAT pathway (signal transducer and transcription activator) is associated with JAK family proteins and is also involved in mediating cellular responses to cytokines. In general, STAT3/5 is involved in the inflammatory and carcinogenic processes, and conversely STAT1 is involved in the inflammatory process [123]. Granato et al. found that quercetin induced autophagy in PEL cells by inhibiting the STAT3 and PI3K/AKT/mTOR signaling pathways. They also indicated that quercetin induced prosurvival autophagy in PEL cells [59].

### 5.9. Role in Apoptosis

In cellular organisms, one of the main causes of changes in cell morphology and eventually cell death is apoptosis or programmed cell death. This process generally occurs through two paths, intrinsic and extrinsic, both of which cause cell death by caspase proteins [124]. During apoptosis, changes occur on the cell surface, including bulging of the cell surface, cell shrinkage, fragmentation of the cell nucleus, chromatin condensation, and mRNA loss [112, 125]. Quercetin stimulates apoptosis through the mitochondrial pathway such as activation of caspase-3 and caspase-9 proteins, the release of cytochrome c, and cleavage of the PARP fragment (poly-ADP-ribose polymerase) [126, 127]. In a study on lymphoma cell lines (U937), quercetin and shHSP27 reduced the expression of proteins involved in the Notch/AKT/mTOR signaling pathway [128]. In another study on the Burji lymphoma cell line (Raji cell line), it was shown that 120 *μ*M of quercetin increased the rate of cell apoptosis [16].

### 5.10. Therapeutic Potential of Quercetin for Lymphoma

Quercetin as an important polyphenolic bioflavonoid that exists in most fruits and vegetables [129] has antimalignant effects besides its antioxidant effects. Recently, some studies indicated that quercetin could suppress the proliferation of cancer cells in vitro [130–132]. Moreover, quercetin has different antimalignant activities against non-Hodgkin's lymphoma including a modulatory effect on the protein kinase (PKC) signaling pathway and apoptosis induction by inhibition of reactive oxygen species and tumor necrosis factor (TNF) receptor, suppression of the STAT3 pathway, and downregulation of Mcl-1, survivin, and p53 [81, 133, 134].

As mentioned above, it has been suggested that the mitochondria are the main targets for quercetin. The treatment with quercetin increased the release following TRAIL stimulation. Quercetin had synergic effects with TRAIL; however, the underlying molecular mechanism has not been identified [135]. The TRAIL-R2 stabilized in quercetin-mediated sensitization to TRAIL. Subsequently the expression of TRAIL-R2 at the cell surface upregulated and increased TRAIL DISC formation [136–138]. Since the mitochondrial apoptotic pathway is suppressed in resistant B-lymphoma cell lines, quercetin could restore the TRAIL apoptotic mechanism [80]. Quercetin-induced restoration of the mitochondrial apoptotic mechanism was correlated with Mcl-1 and survivin dysregulation. Survivin suppresses the mitochondrial Smac release, and it stabilizes XIAP which causes inhibition of caspase-9/3 activation at the postmitochondrial level [139]. Various transcription factors, e.g., signaling pathways such as p53 and AKT, negatively regulate the expression of survivin. In VAL and RL cells, the expression of survivin was inhibited following quercetin stimulation. Further studies are needed to clarify how the expression of survivin was inhibited by quercetin stimulation. Quercetin restores TRAIL-induced apoptosis in aggressive B-lymphoma cell lines via Mcl-1-induced proteasomal degradation. Mcl-1 preserves the cells from TRAIL-induced apoptosis by suppressing Bak, and Bid affects Bax activation. Moreover, quercetin-induced Mcl-1 proteasomal degradation was correlated with Mcl-1 ubiquitination and upregulation. Quercetin at higher concentrations could induce apoptosis via downregulating Mcl-1 and activating Bax [140]. The advantages of quercetin including its low cost, potential beneficial effects, and its pharmacological safety suggest that quercetin could be a promising therapeutic approach for lymphoma.

### 5.11. Limitation of Quercetin Therapy

As mentioned earlier, the plant flavonoid quercetin has frequent biological and pharmacological functions. Numerous studies have shown that quercetin has anti-inflammatory, antioxidant, anticancer, antitoxic, and immunomodulatory properties. In addition to the beneficial and potential properties of quercetin in the treatment of diseases, their use has limitations that researchers need to pay close attention to. These limitations include poor solubility, low bioavailability, hydrophobic nature, and poor permeability. Many researchers are trying to overcome these limitations to use quercetin as a drug with minimal side effects and pharmacological limitations. For example, Nathiya et al. used polymer capsules to increase bioavailability and solubility by placing quercetin inside them [141]. Examining the pros and cons of quercetin in the brain and their diseases, Dajas et al. found that quercetin oxidation leads to the formation of quinones that are not reduced by antioxidants such as tocopherol (vitamin E) and ascorbate (vitamin C) and cause more damage to neurons (neurotoxicity). They concluded that modulating kinases could restore the redox equilibrium and was the only way to reduce quercetin restriction by preventing quinone formation [142]. In addition to these limitations, instability in physiological environments such as the stomach and intestines, a short half-life, and high metabolism in the liver before reaching the bloodstream reduces oral bioavailability. These factors limit the use of quercetin; to overcome these limitations, researchers have proposed efficient drug delivery systems that prevent drug damage to the gastrointestinal tract and reduce its instability when it enters the colon [143–145].

### 5.12. Clinical Studies with Quercetin

Previous studies have shown that quercetin has beneficial effects on many types of diseases. From recent years until now, the clinical (in vivo) use of quercetin in the treatment of various types of cancerous tumors has attracted the attention of many researchers. Several studies have shown that quercetin can exert its anticancer properties through different molecular mechanisms. It has been found that this compound with properties such as cell-cycle arrest, increased apoptosis, inhibition of angiogenesis and metastasis in vivo can help prevent the proliferation of tumor cells and reduce tumor size [146]. For example, in xenograft models of cancers such as leukemia and breast cancer, different doses of quercetin inhibit the cell cycle and increase programmed cell death by inhibiting the AKT/mTOR signaling pathway [147, 148]. In a study on prostate cancer, quercetin inhibited the growth of mouse prostate tumor cells in vivo by increasing the expression of Thrombospondin-1, an antiangiogenic factor [149]. The effect of quercetin on pulmonary-colorectal metastasis was also investigated, and it was found that a dose of 50 mg/kg of this compound reduces tumor metastasis [150]. Another study in BALB/c mice found that a dose of 150 mg/kg quercetin increased the apoptosis of liver tumor cells [151]. In a Phase I clinical trial, Ferry et al. examined the antiproliferative effect of quercetin on cisplatin-resistant ovarian cancer. Following treatment with 420 mg/m^2^, in one patient the level of CA 125 decreased from 295 to 55 units/m^2^; in another patient, the level of serum alpha phytoprotein decreased. As a result, they found that i.v. quercetin inhibits tyrosine kinase activity in lymphocytes and ultimately has an antitumor effect [152]. As a result, due to the fact that quercetin has limited side effects and is well tolerated in humans, it can be used in vivo (clinical trial) to treat a variety of cancers. Unfortunately, clinical studies have not been used to treat lymphoma, so due to the high activity of researchers, it is hoped that this combination can be used to treat lymphoma.

## 6. Conclusion

Quercetin possesses antitumor effects on malignant cells and induces its anticancer effect against cancer cells via modulating various signaling pathways involved in cancer development and progression. Quercetin promotes apoptosis and autophagy by activating caspase-3 and inhibiting AKT, mTOR, and ERK phosphorylation in cancer cells. In addition, quercetin inhibits metastasis by downregulating VEGF and MMP. Quercetin targets mitochondria in cancer cells, decking the bioenergetics and triggering the intrinsic pathway of apoptosis. Quercetin could be helpful as a supplement in cancer prevention and as a low-toxicity therapeutic molecule for cancer treatment. It seems that quercetin can strengthen the effects of other chemotherapeutic drugs. However, more investigations are required to completely elucidate its fully exact mechanisms of action against lymphoid cancer. Taken together, quercetin may be a promising candidate for lymphoid cancer treatment especially in combination with other chemopreventive drugs due to its potential synergistic effects.

## Figures and Tables

**Table 1:** The effects of quercetin in treatment of lymphomas.

	Dose	In vitro/in vivo	Cell line	Effective molecular mechanism	Molecular targets of quercetin	Ref.
Large B-lymphomas	10 or 20 *μ*M	*In vitro*	U937	Arrests cell cycle in the G2/M phase, increases cyclin B protein levels, and decreases cyclin D and E and E2F1 and E2F2 levels	Cyclin B, cyclin D, and cyclin E	[77]
	50 *μ*M	*In vitro*	Daudi and TMD-8	Decreases expression of Notch1 protein	Notch1 protein	[78]
	—	*In vitro*	Namalawa	Arrest of cells in the G2/M phase	Caspase-3	[79]
	20 *μ*M	*In vitro*	VAL, RL, and SUDHL-4 cell lines	Reduces expression of survivin and Mcl-1 and reduces restoration of the TRAIL pathway	TRAIL, Mcl-1	[80]
	20 *μ*M	*In vitro*	DLBCL	Inhibits p-STAT3 and decreases expression of Mcl-1, survivin, and Bcl-x1 genes	STAT3 protein	[81]
	50 *μ*M	*In vitro*	BC1, BC3, and BCBL1	Increases the number of G1 events; increases the inhibition of PI3K and mTOR kinase; reduces the Wnt activation pathway; decreases expression of c-FLIP, cyclin D1, c-Myc, and p65 NF-*κ*B; reduces the release of IL-6 and IL-10; increases expression of HLA II (HLA-DR); decreases expression of HLA I	c-FLIP, cyclin D1, c-Myc, IL-6, IL-10, KSHV proteins, HLA-DR, and calreticulin	[59]
	50 mg	*In vivo*	—	Increases and decreases expression of the *HIGD2A* gene	Higd2a protein	[82]
Burkitt's lymphoma	2.5, 10, and 25	*In vivo*	—	Inhibits primary viral antigens	Ornithine decarboxylase (ODC)	[83]
	30 *μ*M	*In vitro*	Raji cell	Inhibits PI3K*δ*; decreases expression of cIAP-1, cIAP-2, and anti-/proapoptotic molecules; inhibits the mTOR pathway; increases expression of the *γ*H2A.X protein	PI3K and mTOR	[84]
	50 and 100 *μ*M	*In vitro*	Raji, Akata, 2A8, Ramos, and BL-41	Inhibits c-Myc expression and the PI3K/AKT/mTOR pathway, reduces expression of HSP-70, and stimulates autophagy	c-Myc protein, PI3K, AKT, and mTOR	[60]
Multiple myeloma	100 and 200 *μ*mol/L	*In vitro*	NCI-H929	Inhibits CDK4 expression; downregulates p-ERK and p-AKT; activates apoptosis-related proteins caspase-3, caspase-8, caspase-9, and PARP	Caspase-3, caspase-8, caspase-9, PARP, Bcl-2, P53, P21, P27, CDK4, p-ERK, and p-AKT	[85]
	40, 80, 160, and 320 *μ*mol/L	*In vitro*	U266, KM3, RPMI8226, and MM	Downregulates IQGAP1 expression and p-ERK1/2 and inhibits the MAPK pathway in MM cells	IQGAP1 protein and ERK1/2	[86]
T-cell lymphoma	—	*In vivo*	—	Downregulates p-AKT, p-PDK1, p-BAD, p-GSK-3*β*, p-mTOR, p-IkB*α*, VEGF-A COX-2, iNOS, and NO level. Upregulates p-PTEN	AKT, PDK1, BAD, GSK-3*β*, mTOR, IkB*α*, VEGF-A, COX-2, iNOS, and PTEN	[87]
	300 *μ*mol/L	*In vitro*	DLA	Downregulates p-AKT 1, p-PDK1, p-BAD, TNFR1, PKC*α*, and upregulates PTEN	AKT, PDK1, BAD, TNFR1, and PTEN	[88]
